# Chain Modeling of Molecular Communications for Body Area Network

**DOI:** 10.3390/s19020395

**Published:** 2019-01-18

**Authors:** Peng He, Xiaojuan Han, Hanyong Liu

**Affiliations:** 1 School of Communication and Information Engineering, Chongqing University of Posts and Telecommunica-Tions, Chongqing 400065, China; S170101193@stu.cqupt.edu.cn (H.X.); 13028317880@163.com (L.H.); 2 Key Laboratory of Optical Communication and Networks in Chongqing, Chongqing 400065, China; 3 Key Laboratory of Ubiquitous Sensing and Networking in Chongqing, Chongqing 400065, China

**Keywords:** molecular communication, hormonal signaling, Ca^2+^ signaling, neural signaling, chain modeling, body area network

## Abstract

Molecular communications provide an attractive opportunity to precisely regulate biological signaling in nano-medicine applications of body area networks. In this paper, we utilize molecular communication tools to interpret how neural signals are generated in response to external stimuli. First, we propose a chain model of molecular communication system by considering three types of biological signaling through different communication media. Second, communication models of hormonal signaling, Ca${}^{2+}$ signaling and neural signaling are developed based on existing knowledge. Third, an amplify-and-forward relaying mechanism is proposed to connect different types of signaling. Simulation results demonstrate that the proposed communication system facilitates the information exchange between the neural system and nano-machines, and suggests that proper adjustment can optimize the communication system performance.

## 1. Introduction

Rapid progress of nano-technology enables the manufacturing of nano-machines for medical applications of body area network (BAN) [1]. Thus far, a bio-inspired manufacturing method has attracted considerable attention. It assembles biological molecules or atoms into a micro- to nano-scale unit based on the functional structure of nature cells. To finish a complex task, the cooperation of nano-machines is indispensable due to their limited size and power. Molecular communication (MC) is a promising technology for the cooperation of nano-machines, exploiting biological molecules as intra-body information carriers [2]. Design of MC is inspired by mechanisms of biological activities in nature (e.g., hormone communication between ants and neural signaling inside human body), which can be promoted by the experience from diverse traditional communication systems [3,4]. Promising MC applications include health monitoring systems, drug delivery systems and bio-sensor networks [5].

MC could be divided into wireless and wired types based on different properties of biological mechanisms. Existing research efforts generally focus on a single MC type and lack the comprehensive understanding of the complex biological activities in human bodies. Biologically speaking, the human body is a complex system composed of various biological substances including organs, tissues, cells, molecules, etc. It is widely accepted that, even for a simple biological activity, different substances interact with each other through exchange of power and substance. Moreover, processes of biological activities usually involve multiple types of biological signaling, which should be investigated jointly rather than independently. From the aspect of communication, proper integration of bio-systems promote the implementations of complex functionalities. For example, neurons and blood vessels form neurovascular unit, which performs more complex functions via communication pathways, including maintaining the normal activities of neural system, repairing the damaged neurons with the nutrition from the blood, and regulating the vasodilatation of blood vessels [6].

In this paper, we develop a chain model of MC system, based on biological cells, and motivated by different chemical stimuli methods [7]. The model considers three types of MC signaling: hormonal signaling, Ca${}^{2+}$ signaling and neural signaling. Hormonal signaling is based on the diffusion of hormones in fluid medium. Ca${}^{2+}$ signaling is based on the oscillation of Ca${}^{2+}$ ions in astrocytes. Neural signaling is based on the spike train transportation of action potential in neurons. The proposed model involves a chain of communications, in which hormonal signals are considered as the input, triggering oscillation of Ca${}^{2+}$ signals. As the relay, Ca${}^{2+}$ signals further induce neural signals as the output. The proposed chain model relies on different bio-sensors in biological cells. For example, hormones are observed by G-protein bio-sensors in astrocytes (see Section 5), and neural signals are collected by AMPA or NMDA bio-sensors in neurons (see Section 6).

This paper is developed based on our earlier work [8], where an initial hybrid model of MC is proposed including diffusive and neural channels. In that work, nano-machines are grouped to function as the sender, relay and receiver. The communication model in [8] includes high biological plurality and therefore is reliable. Novel contributions of this paper are listed as follows.

- First, we develop a chain model of molecular communication based on biological signaling. The proposed model considers the biological interactions among hormone, Ca${}^{2+}$ and neural signals, which is more general than the model of [8].
- Second, we propose an implementable amplify-and-forward relaying mechanism instead of decode-and-forward relaying as in [8]. In addition, multiple astrocytes are utilized instead of a nano-machine, elevating the reliability of relaying from Ca${}^{2+}$ signaling to neural signaling.
- Third, based on the work in [8], we examined the relations between communication performance and more parameters of the proposed model. We also found that source coding is efficient in improving the communication performance, which may provide a guidance for nano-machine design.

## 2. Related Work

We examined the literature on MC related to this work. Diffusion-based MC has attracted great attention due to its universality in biology. In [9], a mathematical model of communication is developed based on Brownian motion, and the channel capacity is theoretically deduced. In [10], a joint optimization strategy is developed to improve the performance of the two-hop diffusion-based MC system. In [11], different constant composition codes are designed for channel detection at the receiver without channel state information.

Another potential type of MC is Ca${}^{2+}$ signaling. In [12], a linear channel model for intra/inter-cellular Ca${}^{2+}$ MC system is developed, where the channel gain and communication delay are analyzed. In [13], Ca${}^{2+}$ signaling models for different types of cells are introduced and compared. In addition, various communication performance indicators are deduced for these models. In [14], a channel switching functionality is designed using Ca${}^{2+}$ signaling in three biological cells, and different gating properties of gap junction channels are considered.

Neural signaling has been abundantly studied based on the knowledge of neural science. What concerns us most is the communication model in neural networks, and there are innumerable studies focusing on this area. In [15], a multi-input-single-output model of synaptic channel in hippocampus area of the brain is developed, and the communication performance is analyzed using Shannon theory. In [16], the capacity of the axonal channel is derived, where parameters are optimized to improve the performance. In [17], a fast algorithm is proposed for performance analysis of artificial and biological neural systems.

## 3. Overview of the Communication System

**Figure 1:**
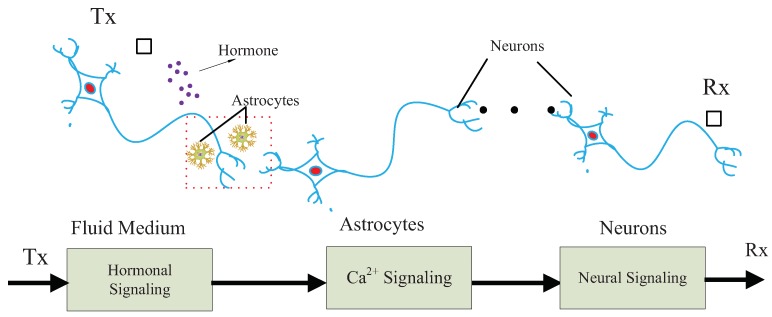
Proposed chain of MC system containing hormonal, Ca${}^{2+}$ and neural signaling.

The proposed MC system is illustrated in Figure 1. The proposed communication system is motivated by the experience of multiple traditional communication systems [18,19,20,21,22,23]. Specifically, the communication system is modeled as a chain, which is composed of a sender, a receiver and three types of communication media. The sender and receiver are nano-machines with different functions, respectively, denoted by Tx and Rx. Tx is assumed to release hormones in response to external stimuli. Tx can be, for example, engineered endocrine cells [2], which are able to release hormones under control. Rx is a bio-inspired or electronic nanomachine that is able to detect neural signals.

The first type of communication medium is the fluid, where hormones propagate around the astrocytes. There are two types of propagation mechanisms, determined by types and properties of the hormones and fluid medium. First, hormones perform approximative random walk towards any direction with low energy consumption in the passive diffusion mechanism. For example, steroid hormones propagate in the brain via lipid mediator and passive diffusion [24]. Second, hormones drift towards specific directions with high energy consumption in active diffusion. For example, thyroid hormones permeate blood–brain barrier by a slow flux rate in bloodstream via active diffusion [25].

The second type of communication medium is astrocytes, which are specific cells connected with neurons. Astrocytes are closely related to neural activities, such as physically supporting structures of neurons, providing nutrition for neurons, and promoting the growth of neurons [26]. Astrocytes can promote the generation of neural signals through adjustment of Ca${}^{2+}$ oscillation, thereby serving as nature bridges between another biological system and neural system. Reportedly, astrocytes can respond to hormones (e.g., norepinephrine) in generation of Ca${}^{2+}$ signals [27]. Based on this knowledge, astrocytes function as the perfect relay in our proposed model, connecting hormonal signaling with neural signaling.

The third type of communication medium is neuron, which processes and transmits information through the human body. Neural communication is an efficient and reliable biological signaling, exploiting both electrical and chemical methods. Different types of neurons respond to different external stimuli (e.g., electricity, chemical and light) [28].

As shown in Figure 1, the communication system is developed to interpret the neural signaling evoked by chemical stimuli. We assume that the proposed biological communication system transmits information by encoding the presence or absence of signals in time scale. Moreover, we assume that time-slotted mechanism works for the proposed communication system, based on the biological clocks guaranteeing the synchronization of biological cells [29].

The communication process is described as follows. Initially, Tx releases hormones in a controlled pattern, responding to external stimuli. Then, hormones diffuse in the fluid medium, some of which are absorbed by the astrocytes, inducing the oscillation of Ca${}^{2+}$ signaling. Oscillated Ca${}^{2+}$ ions in astrocytes flow into the neurons and trigger neural signaling. Finally, the neural signals are detected and decoded by Rx.

## 4. Hormonal Signaling Model

In Tx, a list of binary information is encoded with the number of released hormone molecules per time slot. One bit is transmitted in a symbol period, denoted by *T*. It is found that secretion pattern of hormones is usually with burst and not sustaining [30]. Accordingly, we apply a simple coding for MC, i.e., on–off key (OOK) with square pulses. During a time of *T*, a certain number of hormone molecules $Q_{0}$ are released into fluid medium, if Tx transmits bit “1”; no hormone molecules are released into fluid medium, if Tx transmits bit “0”. Let $R_{H}\left(t\right)$ be the released molecules during the *i*th slot, the expression of OOK coding is given by,

(1) $$R_{H}\left(t\right)=\left\{\begin{array}{cc} Q_{0}\delta \left(t-iT\right) & \mathrm{bit}\;=\;“1”\\ 0 & \mathrm{bit}\;=\;“0” \end{array}\right.$$

where δ(t) is the Dirac’s delta function of *t*. Hormone molecules propagate with random or directed walks, motivated by the passive or active diffusion mentioned in Section 3. Plenty of factors impact the distance and velocity of diffusion processes, including molecules diameter, medium type, temperature and pressure. For simplicity, we only consider passive diffusion of hormones, i.e., hormones performing random walks. Concentration of the hormone molecules presents a decreasing gradient with the distance, which could be expressed by the well-known Fick’s second law,

(2) $$\frac{dC_{H}\left(x,t\right)}{dt}=D_{H}\nabla^{2}C_{H}\left(x,t\right)+R_{H}\left(t\right)$$

where $\nabla^{2}$ is the Laplace operator, indicating the sum of the second derivatives of $C_{H}\left(x,t\right)$. $R_{H}\left(t\right)$ is the releasing rate of molecules at Tx in time scale. $D_{H}$ is the diffusion coefficient of hormones, indicating the diffusion rate. Let $x_{S}$ be the position of Tx, and $x_{A}$ denote the position of an astrocyte. By solving Equation (2), the concentration accumulated at the position of $x_{A}$ is given by,

(3) $$\begin{array}{cc} C_{H}\left(x_{A},t\right)= & R_{H}\left(t\right)\cdot g\left(d_{S,A},t\right)\\ = & \frac{R_{H}\left(t\right)}{{\left(4\pi D_{H}t\right)}^{\frac{3}{2}}}exp\left(-\frac{d_{S,A}^{2}}{4D_{H}t}\right) \end{array}$$

where g(x,t) is the Green function of hormones [9], expressing the concentration distribution of hormone molecules in space and time. $d_{S,A}$ denotes the distance between Tx and an astrocyte, given by $d_{S,A}=\left|x_{A}-x_{S}\right|$. A single hormonal molecule could be released by Tx in the $n_{S}$th slot and absorbed by an astrocyte in the $n_{A}$th slot. The corresponding probability is given by [31],

(4) $$u_{n}=\int_{nT}^{(n+1)T}g\left(d_{S,A},t\right)\int_{t}^{\infty }m_{H}\left(v\right)dvdt$$

where $n=n_{A}-n_{S}$, and $m_{H}\left(v\right)$ denotes the probability function distribution of the molecular life expectancy, given by $m_{H}\left(v\right)=\iota e^{-\iota v}$ with mean of 1/ι.

## 5. Calcium Signaling Model

Astrocytes are special star-shaped glial cells, physically close to neurons, and play a crucial role in regulation of neural activities. It is observed that astrocytes propagate Ca${}^{2+}$ waves in response to external stimuli, which further induce and impact the behaviors of neural signaling [32]. In this section, we explain how Ca${}^{2+}$ signals are generated and propagate in astrocytes.

**Figure 2:**
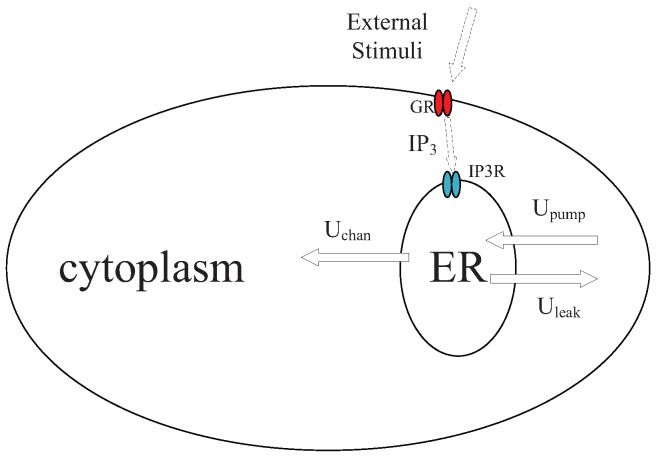
Dynamic of Ca${}^{2+}$ signals in an astrocyte.

We introduce a mathematical model of Ca${}^{2+}$ signaling in astrocytes according to experimental observations [27]. The dynamic of Ca${}^{2+}$ signaling in an astrocyte is shown in Figure 2. One major organelle that stores Ca${}^{2+}$ ions is endoplasmic reticulum (ER). With an external stimulus in astrocyte, Inositol 1,4,5-trisphosphate (IP${}_{3}$) molecules are generated by G-protein coupled receptors (GR), changing the permeability of IP${}_{3}$-sensitive Ca${}^{2+}$ receptors (IP${}_{3}$R) of the ER and induce the Ca${}^{2+}$ fluxes. Three major Ca${}^{2+}$ fluxes are considered, namely channel fluxes released from the ER to the cytoplasm via receptors of IP${}_{3}$ ($U_{chan}$), uptake fluxes from the cytoplasm to the ER by Ca${}^{2+}$ pumps ($U_{pump}$), and leak fluxes from from the ER to the cytoplasm ($U_{leak}$). Induced Ca${}^{2+}$ fluxes incur the oscillation of Ca${}^{2+}$, and patterns of Ca${}^{2+}$ waves are positively related to the concentration of external stimuli. Let $C_{CY}$ and $C_{ER}$ denote the Ca${}^{2+}$ concentrations in cytoplasm and ER. Variable $h_{\infty }$ is employed to denote the Ca${}^{2+}$ channel inactivation gate. On the time scale, the introduced model of Ca${}^{2+}$ signaling is expressed as a set of equations,

(5) $$\frac{dC_{CY}}{dt}=a_{1}\left(U_{chan}+U_{leak}\right)-U_{pump}$$

(6) $$\frac{dC_{ER}}{dt}=U_{chan}+U_{leak}-\frac{1}{a_{1}}\cdot U_{pump}$$

(7) $$\frac{dh_{\infty }}{dt}=\frac{a_{3}-h_{\infty }}{a_{2}}$$

where,

(8) $$U_{chan}=v_{chan}\cdot {\left(\frac{C_{IP3}}{C_{IP3}+b_{1}}\right)}^{3}{\left(\frac{C_{CY}}{C_{CY}+b_{2}}\right)}^{3}h_{\infty }^{3}\left(C_{ER}-C_{CY}\right)$$

(9) $$U_{leak}=v_{leak}\cdot \left(C_{ER}-C_{CY}\right)$$

(10) $$U_{pump}=v_{pump}\cdot \frac{{C_{CY}}^{2}}{{C_{CY}}^{2}+{b_{3}}^{2}}$$

(11) $$a_{2}=\frac{1}{b_{6}\left(b_{f}+C_{CY}\right)}$$

(12) $$a_{3}=\frac{b_{f}}{b_{f}+C_{CY}}$$

(13) $$b_{f}=b_{5}\frac{C_{IP3}+b_{1}}{C_{IP3}+b_{4}}$$

In the above equations, $a_{1}$ denotes the ratio of ER to cytoplasm, $a_{2}$ denotes the time constant of the inactivation gate, and $a_{3}$ denotes the steady value of the inactivation gate. $v_{chan}$, $v_{leak}$ and $v_{pump}$ denote the maximum flux rates of $U_{chan}$, $U_{leak}$ and $U_{pump}$, respectively. $b_{1}$–$b_{6}$ are constants of the model, and $C_{IP3}$ denotes the concentration of IP${}_{3}$.

On the spatial scale, the propagation of Ca${}^{2+}$ ions in astrocytes is based on the diffusion of Ca${}^{2+}$ [12]. ER is modeled as a point source of releasing Ca${}^{2+}$. Ca${}^{2+}$ ions around the boundary of astrocytes can flow into the connected neurons. For any position *x* inside an astrocyte, the dynamic of the Ca${}^{2+}$ concentration via diffusion can be expressed by,

(14) $$\frac{dC_{CY}\left(x,t\right)}{dt}=D_{Ca}\Delta C_{CY}\left(x,t\right)+R_{Ca}\left(t\right)$$

where $D_{Ca}$ denotes diffusion coefficient of Ca${}^{2+}$ smf $R_{Ca}\left(t\right)$ denotes the variation rate of Ca${}^{2+}$ concentration, determined by Equations (5)–(13). Equation (14) can be solved similarly to Equation (3).

## 6. Neural Signaling Model

Neural communication is a fast biological signaling, and its velocity varies due to different neuron types. Neural communication is also biologically reliable, as the neurons are able to eliminate errors in communications. In this section, we introduce a signaling model across two neighboring neurons, including the processes of neural firing, axonal transmission, gap junctional transmission, and postsynaptic response.

One neuron is normally composed of different number of dendrites, axons, somas, and axonal terminals. In a communication, neural signals are first generated by ions through neural firing process in soma, transmitted along the axon fiber in the pattern of electrical impulses. Then, the electrical impulses trigger the release of neurotransmitters from the vesicles located in axon terminals. The neurotransmitters propagate to the neighboring neurons, until they are absorbed by the dendrites. Finally, the postsynaptic response is induced that might trigger firing of neighboring neurons.

### 6.1. Amplify-and-Forward Neural Firing

The generation of neural signals is called neural firing, which is an all-or-none process. Various types of ions including Ca${}^{2+}$ are released by astrocytes and flow into neurons. During the process, local potentials of neuron membrane are gradually elevated and aggregated into a global potential. If the global potential exceeds a threshold, neural signals are generated. Otherwise, it fails to generate neural signals since the global potential falls quickly.

Neural firing is a relaying process, in which information carrier varies from Ca${}^{2+}$ to neural signals. In our model, we apply amplify-and-forward (AF) relaying in neural firing process. AF relaying is based on the concentration-mediated mechanism in astrocyte–neuron communications [33]. We assume that local neural signals are positively elevated according to the concentration of Ca${}^{2+}$ without decoding.

To generate neural signals, there must be adequate Ca${}^{2+}$ ions flowing into neurons. The number of astrocytes should be sufficient to generate the required intensities of Ca${}^{2+}$ flows. The local potentials are elevated by Ca${}^{2+}$ from connected astrocytes, which cooperate for achieving neural firing. This process is expressed by the well-known Hodgkin–Huxley model [34], given by,

(15) $$V_{N}\left(t\right)=V_{r}+\sum_{i}^{M_{A}}\int_{t_{i}}^{\infty }\nu \left(s\right)A_{N,i}\left(t-s\right)ds$$

where $M_{A}$ is the total number of astrocytes contributing to the neural firing, $V_{N}\left(t\right)$ denotes the global potential on the neural membrane during the firing process, $V_{r}$ is the resting global potential without any stimuli, ν is the linear response of the neural membrane to an input pulse of Ca${}^{2+}$ flow, $A_{N,i}\left(t\right)$ denotes the intensity of Ca${}^{2+}$ flow released by the *i*th astrocyte, injected into the neuron at time $t_{i}$, and $A_{N,i}\left(t\right)$ is proportional to Ca${}^{2+}$ concentration of astrocyte, expressed by $A_{N,i}\left(t\right)\propto C_{CY}\left(t\right)$. The action potential spikes are typical neural signal S(t), generated when $V_{N}\left(t\right)$ is strong enough to exceed firing threshold $\theta_{1}$,

(16) $$S\left(t\right)=\sum_{j}\varphi \left(t-t_{j}\right)$$

where φ(t) denotes the waveform of action potential spike, and $t_{j}$ denotes the time that the spike occurs. Based on experimental observations, S(t) follows or approaches Poisson distribution in response to random intensities of Ca${}^{2+}$ flows [35]. We employ λ(t) to denote the firing rate, which is determined by not only the Ca${}^{2+}$ flows, but also the inherent capability of neurons. The distribution of S(t) could be impacted by the noise or some other stochastic factors.

### 6.2. Axonal Transmission

In the process of axonal transmission, action potential spikes transmit along the axon until they arrive in the axon terminals. During the process, various ions (e.g., $K^{+}$, $Na^{+}$ and $Cl^{-}$) exchange between the inside and outside of the axon fiber. The axonal transmission process could be modeled by the cable theory, expressed by the quantitative equation in [36],

(17) $$\frac{\gamma_{m}}{\gamma_{l}}\frac{d^{2}V_{N}\left(x,t\right)}{dx^{2}}=\gamma_{c}\gamma_{m}\frac{dV_{N}\left(x,t\right)}{dt}+V_{N}\left(x,t\right)$$

where $\gamma_{m}$, $\gamma_{l}$ and $\gamma_{c}$ denote the membrane resistance, longitudinal resistance and capacitances, respectively. $V_{N}\left(x,t\right)$ denotes the potential of axonal fiber at time *t* and location *x*. The boundary condition of the axonal transmission is given by,

(18) $$\left\{\begin{array}{c} V_{N}\left(x,0\right)=V_{0}\left(x\right)\\ V_{N}\left(0,t\right)=0 \end{array}\right.$$

The above equations can be solved using the Fourier method [37], namely $V_{N}\left(x,t\right)$ is calculated as,

(19) $$V_{N}\left(x,t\right)=exp\left(\frac{t}{-\gamma_{m}\gamma_{c}}\right)\sum_{i=1}^{\infty }\zeta_{i}exp\left[-\frac{\pi^{2}i^{2}t}{{L_{N}}^{2}r_{l}\gamma_{c}}\right]cos\left(\frac{\pi \cdot i\cdot x}{L_{N}}\right)$$

where $L_{N}$ is the length of the axon fiber. $\zeta_{i}$ denotes the Fourier coefficient, following the boundary conditions in Equation (18), and can be obtained by,

(20) $$\zeta_{i}=\frac{2}{L_{N}}\int_{0}^{L_{N}}V_{0}\left(x\right)cos\left(\frac{\pi \cdot i\cdot x}{L_{N}}\right)dx$$

### 6.3. Gap Junctional Transmission

#### 6.3.1. Vesicle Release

**Figure 3:**
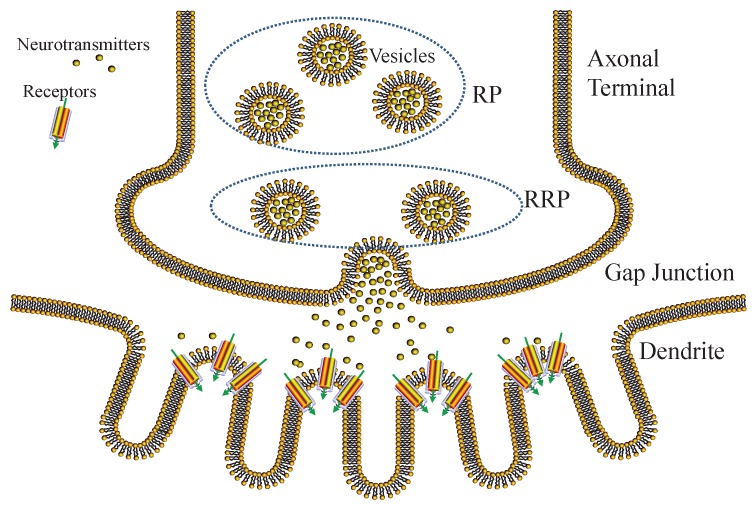
Diagram of gap junctional transmission.

We show the process of gap junctional transmission in Figure 3. Action potential spikes arrive in the axonal terminals, triggering vesicle movements and inducing vesicle-membrane fusion. As a result, neurotransmitters stored inside vesicles are released into the gap junction. The release process of neurotransmitters is stochastic, related to the vesicle-membrane distance [38]. Therefore, we exploit a pool model to classify vesicles into readily releasable pool (RRP) and reserve pool (RP), respectively. The vesicles of RP are difficult to release, since they are far from the membrane, whereas the vesicles of RRP are easy to release, since they are close to the membrane.

Let $N_{RRP}$ and $N_{RP}$, respectively, denote the sizes of RRP and RP. The vesicles of RP are assumed to fill the RP with rate of $\tau_{f}$, if $N_{RRP}$ is reduced due to the release process. The average firing rate of vesicles is denoted by $\bar{\lambda }$, which is calculated as the integral of firing rate λ(t) during a time slot, 

(21) $$\bar{\lambda }=\frac{\int_{0}^{T}\lambda \left(t\right)dt}{T}$$

The release probability of a vesicle is related to $\bar{\lambda }$ and $N_{RRP}$, given by,

(22) $$P_{rele}=1-exp\left(\bar{\lambda }N_{RRP}\right)$$

In local scale, $P_{rele}$ can be approximated as the linear relation with $\bar{\lambda }$, i.e., $P_{rele}=\bar{\lambda }N_{RRP}$. Due to the stochastic behavior of neurotransmitters, it is possible to cause errors during the vesicle release process. For binary information, the error probabilities of bit “1” and “0” are, respectively, calculated by,

(23) $$P_{e\_neuron_{1}}^{\left(1\right)}=1-{\left(P_{rele}\right)}^{k_{i}}$$

(24) $$P_{e\_neuron_{1}}^{\left(0\right)}=P_{rele}^{k_{i}}$$

where $k_{i}$ is the number of action potential spikes during the *i*th slot. Equations (23) and (24) indicate that error transmission of binary information is caused by miss or redundant release of vesicles.

#### 6.3.2. Queueing Model of Neurotransmitters

The neurotransmitters diffuse in the gap junctions until absorbed by the dendrite receptors of neighboring neurons. Due to the stochastic behavior of the vesicle release process, the absorbed time of neurotransmitters is random. The diffusion of neurotransmitters is slow; this process is normally ignored since gap junctions are extremely short (several nanometers). However, neurotransmitters can be absorbed with delay due to the limited absorbing ability of dendrites [39]. Generally, neurotransmitters gather around the receptors of dendrites and wait to be absorbed. To study this phenomenon, we use the queuing theory to calculate the delay of gap junctional transmission.

In our model, the neurotransmitters are defined as the batched customers, which are independent with identical distributions. We adopt the $M^{x}|G|1|K$ model, where $M^{x}$ is the absorbing time of these batched neurotransmitters. The time intervals of vesicle release follow the negative exponential distribution, i.e., $f\left(t_{i}\right)=\sigma \lambda_{b}exp\left(-\sigma \lambda_{b}t_{i}\right)\left(0<\sigma <1\right)$. $\mu_{b}$ and $\tau_{b}$, respectively, denote the batch size and batch service rate, satisfying $\lambda_{b}=\mu_{b}\tau_{b}$. *K* denotes the capacity of the receptor. *G* denotes absorbing time $t_{s}$ of the receptor with a distribution G(t), which is given by $0<t_{s}=\frac{1}{\mu }=\int_{0}^{\infty }tdG\left(t\right)<\infty$. The distribution of the batch sizes is expressed as $\epsilon \left(x\right)={\left(1-\sigma \right)}^{x-1}\sigma$. Based on the result of [40], the blocking probability is given by,

(25) $$P_{blo}=p_{T}\left(0\right)\frac{\tau_{b}{\left(1-\sigma \right)}^{0}}{\tau_{b}\left(1-\sigma \right)+\sigma }+\sum_{t=1}^{N-1}p_{T}\left(t\right){\left(1-\sigma \right)}^{\left(N-t\right)}+p_{T}\left(N\right)$$

where $p_{T}\left(k\right)$ is the probability that neurotransmitters are absorbed by *k* customers. The blocking phenomenon is more serious for bigger $\lambda_{b}$. Based on the previous derivations, the average delay is calculated as,

(26) $$\begin{array}{cc} \omega = & \frac{\sum_{k=1}^{N-1}\sum_{r=i}^{\infty }\sum_{i=1}^{N-k}p_{T}\left(t\right)\epsilon \left(r\right)\int_{0}^{\infty }g_{0}\left(k+i-1,t\right)dt}{\epsilon (1-P_{blo})}\\ & +\frac{\sum_{r=i}^{\infty }\sum_{i=1}^{N}p_{T}\left(0\right)\epsilon \left(r\right)\int_{0}^{\infty }g_{0}\left(i-1,t\right)dt}{\epsilon (1-P_{blo})} \end{array}$$

where $g_{0}\left(x,t\right)$ is the probability that customers are served at time *t*.

### 6.4. Postsynaptic Response

The process of postsynaptic response includes the formation of postsynaptic potential and the decoding of neural signals.

#### 6.4.1. Postsynaptic Potential

The neurotransmitters are absorbed by dendrites of neighboring neurons via various receptors, such as the AMPA and NMDA receptors. Neurotransmitters accumulate on the dendrite membrane, namely postsynaptic terminals. During this process, the postsynaptic potential of dendrite membrane is elevated to generate the waveform of output signals, which could be expressed by an alpha function [41],

(27) $$ϝ\left(t\right)=G_{max}\frac{t}{t_{p}}exp\left(1-\frac{t}{t_{p}}\right)$$

where $G_{max}$ is the maximum amplitude of the potential, and $t_{p}$ is the corresponding time to peak.

#### 6.4.2. Neural Decoding

Decoding process of the neural signal includes two steps: (1) estimation of the action potential spikes; and (2) extraction of the binary information.

In the first step, a waveform of output signal is denoted by y(t), which could be calculated as a convolution of the action potential spikes and ϝ(t),

(28) $$y\left(t\right)=q\sum_{i}\delta \left(t-t_{i}\right)\cdot ϝ\left(t\right)$$

where *q* denotes a stochastic variable in the signal amplification, following the Gamma distribution [42]. To extract the binary information of y(t), we first sample y(t) into discrete signal y(n) with interval $\tau_{s}$,

(29) $$y\left(n\right)=\sum_{j=-N_{sam}/2}^{N_{sam}/2}y\left(n-j\tau_{s}\right)$$

where $N_{sam}$ is the number of samplings in waveform of y(t), which depends on $G_{max}$ and $t_{p}$. Let $t_{p}$ be the origin point; $\frac{N_{sam}}{2}$ sampling points are determined on both sides of the origin point, where $k\tau_{s}<\min\left(t_{j}-t_{j-1}\right)$ is satisfied. The sampling interval $\tau_{s}$ should be properly regulated to alleviate the distortion of sampling signals. We adopt threshold $\vartheta_{2}$ to determine whether a spike exists or not at the position of sampling. If $\frac{\sum_{n=1}^{k}y\left(n\right)}{k}<\vartheta_{2}$, a spike exists. Accordingly, we obtain a pattern of estimated spikes, denoted by $y^{\prime }\left(t\right)$.

In the second step, we extract the binary information by examining the distribution of $y^{\prime }\left(t\right)$. The Kolmogorov–Smirnov test [43] is employed to verify whether the distribution of $y^{\prime }\left(t\right)$ follows the Poisson distribution in time slot *T*. We say $y^{\prime }\left(t\right)$ follows the Poisson distribution if the following condition is satisfied,

(30) $$\sqrt{i}\varkappa_{i}>K_{\alpha }$$

where variable $K_{\alpha }$ meets condition $P(K⩽K_{\alpha })=1-\alpha$, *K* denotes the Kolmogorov distribution, and $\varkappa_{i}$ is the Kolmogorov–Smirnov statistic of the Poisson process during the *i*th slot. We obtain $\lambda^{\prime }$ using the Maximum Likelihood Estimation (MLE). Threshold $\vartheta_{3}$ is employed for decoding, which is smaller than λ. For the *i*th bit, if the condition in Equation (30) is satisfied and estimated $\lambda^{\prime }$ is higher than $\vartheta_{3}$, the decoded information is bit “1”. Otherwise, the decoded information is bit “0”. Further, we deduce the transmission error probabilities of decoding for both bit “1” and “0”,

(31) $$P_{e\_neuron_{2}}^{\left(0\right)}=P\left(\sqrt{i}\varkappa_{i}>K_{\alpha }\right)P\left(\lambda^{\prime }⩾\vartheta_{3}\right)$$

(32) $$P_{e\_neuron_{2}}^{\left(1\right)}=P\left(\sqrt{i}\varkappa_{i}⩽K_{\alpha }\right)+P\left(\sqrt{i}\varkappa_{i}>K_{\alpha }\right)P\left(\lambda^{\prime }<\vartheta_{3}\right)$$

According to Equations (23), (24), (31) and (32), the error probability of transmitting one bit neural signal is expressed as,

(33) $$\begin{array}{cc} P_{neuron}\left(1|0\right)= & P\left(bit=“0”\right)P_{e\_neuron_{1}}^{\left(0\right)}\left(1-P_{e\_neuron_{2}}^{\left(1\right)}\right)\\ & +p\left(bit=“0”\right)\left(1-P_{e\_neuron_{1}}^{\left(0\right)}\right)P_{e\_neuron_{2}}^{\left(0\right)} \end{array}$$

(34) $$\begin{array}{cc} P_{neuron}\left(0|1\right)= & P\left(bit=“1”\right)P_{e\_neuron_{1}}^{\left(1\right)}\left(1-P_{e\_neuron_{2}}^{\left(0\right)}\right)\\ & +p\left(bit=“0”\right)\left(1-P_{e\_neuron_{1}}^{\left(1\right)}\right)P_{e\_neuron_{2}}^{\left(1\right)} \end{array}$$

## 7. Channel Capacity and Transmission Delay

In this section, we deduce the expressions of channel capacity and transmission delay for the proposed communication system.

### 7.1. Channel Capacity

Let $X_{i}$, $Y_{i}$ and $Z_{i}$, respectively, represent the input, relay and output signal, indicating the diffusive concentration of hormone, oscillating concentration of Ca${}^{2+}$, and decoded action potential spikes. To deduce the channel capacity, we define the following equations,

(35) $$P_{i}^{\left(1,1\right)}=P\left[Y_{i}=0|X_{i}=0\right]$$

(36) $$P_{i}^{\left(1,2\right)}=P\left[Y_{i}=1|X_{i}=1\right]$$

(37) $$P_{i}^{\left(2,1\right)}=P\left[Y_{i}=0|X_{i}=0\right]P\left[Z_{i}=0|Y_{i}=0\right]$$

(38) $$P_{i}^{\left(2,2\right)}=P\left[Y_{i}=1|X_{i}=1\right]P\left[Z_{i}=1|Y_{i}=1\right]$$

(39) $$P_{i}^{\left(2,3\right)}=P\left[Y_{i}=0|X_{i}=1\right]P\left[Z_{i}=1|Y_{i}=0\right]$$

(40) $$P_{i}^{\left(2,4\right)}=P\left[Y_{i}=1|X_{i}=0\right]P\left[Z_{i}=0|Y_{i}=1\right]$$

In the above equations, $P_{i}^{\left(1,1\right)}$ and $P_{i}^{\left(1,2\right)}$ are related to the probability of transmitting bit “1”, denoted by *p*. According to the conclusions in [31], the expressions of $P_{i}^{\left(1,1\right)}$ and $P_{i}^{\left(1,2\right)}$ are, respectively, given by,

(41) $$P_{i}^{\left(1\right)}=\prod_{i=1}^{N_{b}-1}\left(1-pu_{i}\right)$$

(42) $$Q_{i}^{\left(1\right)}=1-\left(1-u_{0}\right)\prod_{i=1}^{N_{b}-1}\left(1-pu_{i}\right)$$

where $u_{i}$ is the probability of Equation (4), and $N_{b}$ is the total number of time slots. The expressions of $P[Z_{i}=1|Y_{i}=0]$ and $P[Z_{i}=0|Y_{i}=1]$ can be calculated according to Equations (33) and (34). Based on information theory, the channel capacity is expressed as,

(43) $$C=max\sum_{i=1}^{N_{b}}\frac{I(X_{i};Z_{i})}{N_{b}}$$

We assume that, under the condition of Y, X is uncorrelated to Z. During the *i*th time slot, the mutual information is given by,

(44) $$\begin{array}{cc} I(X_{i};Z_{i}) & =I\left(X_{i};Y_{i}Z_{i}\right)-I\left(X_{i};Y_{i}/Z_{i}\right)\\ & =I\left(X_{i};Y_{i}\right)-I\left(X_{i};Y_{i}/Z_{i}\right) \end{array}$$

The mutual information between X and Y is further calculated as,

(45) $$\begin{array}{cc} I(X_{i};Y_{i})= & H\left(Y_{i}\right)-H\left(Y_{i}|X_{i}\right)\\ = & \Psi (\left(1-p\right)P_{i}^{\left(1,1\right)}+p\left(1-P_{i}^{\left(1,2\right)}\right))\\ & -p\Psi \left(P_{i}^{\left(1,2\right)}\right)-\left(1-p\right)\Psi \left(P_{i}^{\left(1,1\right)}\right) \end{array}$$

where the Ψ(x)=−xlogx−(1−x)log(1−x). The second item of Equation (44) is further expanded as,

(46) $$\begin{array}{cc} I(X_{i};Y_{i}/Z_{i}) & =H\left(X_{i}|Z_{i}\right)-H\left(X_{i}|Y_{i}Z_{i}\right)\\ & =-H\left(Y_{i}Z_{i}|X_{i}\right)+H\left(Y_{i}Z_{i}\right)+H\left(Z_{i}|X_{i}\right)-H\left(Z_{i}\right) \end{array}$$

### 7.2. Delay

Transmission delay of the proposed communication system is denoted by Ω, which is calculated as the delay summation of hormonal signaling $\Omega_{H}$, Ca${}^{2+}$ signaling $\Omega_{Ca}$ and neural signaling $\Omega_{N}$,

(47) $$\Omega =\Omega_{H}+\Omega_{Ca}+\Omega_{N}$$

The estimation of $\Omega_{H}$ is related to the emission, propagation and reception processes of hormones. For simplicity, we only consider the propagation process in channel for the transmission delay calculation. The transmission delay is related to two factors on the scales of time and space. The first factor is the transmission distance on the spatial scale. One feasible method is to estimate based on the probability distribution function along propagation distance $d_{S,A}$, given by,

(48) $$\begin{array}{cc} f_{\Omega_{H}}\left(d_{S,A}\right)= & \int_{0}^{d_{S,A}}g\left(x,t\right)dx\\ = & \frac{erf(\sqrt{\frac{d_{S,A}^{2}}{4D_{H}\Omega_{H}}})}{8\pi D_{H}\Omega_{H}} \end{array}$$

where erf(x) is the error function, expressed as $erf\left(x\right)=\frac{2}{\sqrt{\pi }}\int_{0}^{x}e^{-u^{2}}du$. Another factor determining transmission delay is the variation rate of molecules at the sender on the time scale. Due to the fixed number of molecules $Q_{0}$ in one time slot, the variation rate of molecules is determined by the length of each time slot. The average delay of hormonal diffusion is calculated as,

(49) $$\Omega_{H}\left(d_{S,A}\right)=\int_{0}^{T}\Omega_{H}f\left(\Omega_{H}\right)d\Omega_{H}$$

The estimation of $\Omega_{Ca}$ is based on the time difference, which starts with the generation of Ca${}^{2+}$ waves in connected astrocytes, ends with the absorbing of Ca${}^{2+}$ by a neuron. Similar with the estimation of Taynnan et al. [13], the variational concentrations of Ca${}^{2+}$ in the astrocytes are assumed to be equal with the absorbed concentrations of Ca${}^{2+}$ during an increasing delay of $\Omega_{Ca}$ [13], i.e.,

(50) $$\sum_{i=1}^{M_{A}}\int_{0}^{T}\Delta (C_{CY}^{i}\left(t\right))dt=\int_{0}^{T+\Omega_{Ca}}\Delta (C_{N}\left(t\right))dt$$

where Δ(x) denotes the variation of *x* during a time period, $C_{CY}^{i}\left(t\right)$ denotes the Ca${}^{2+}$ concentration in cytoplasm of the *i*th connected astrocyte with the neuron, and $C_{N}\left(t\right)$ is the variational concentration of Ca${}^{2+}$ absorbed by the neuron.

In the calculation of the neural signaling delay, we mainly consider the contribution of gap junctional transmission. Based on the proposed queuing model of gap junctional transmission, the average delay of the vesicle release process is approximated as the average waiting time ω of the queueing model. The delay of axonal transmission $\Omega_{Ax}$ is calculated based on Equation (19). Let $N_{N}$ be the number of neurons, and the average delay between two neighboring neurons is expressed as, 

(51) $$\Omega_{N}=N_{N}\left(\omega +\Omega_{Ax}\right)$$

## 8. Performance Evaluation

A computer simulation was employed to examine the performance of proposed communication system. The control variate method was exploited since there are too many parameters in the proposed system. We verified the relations between some important parameters and the performance indicators (i.e., channel capacity and transmission delay).

### 8.1. Simulation Design

Simulation parameters were grouped according to hormonal, Ca${}^{2+}$ and neural signaling, respectively.

Parameters of the hormonal signaling are listed in [9,31,44]: diffusion coefficient $D_{H}$ was fixed at 4.8 μm${}^{2}$/ms, number of released molecules per bit $Q_{0}$ varied from 1000 to 10,000, the distance between Tx and astrocytes were randomly generated within 1–20 μm, the length of time slot *T* varied from 1 s to 20 s, molecule life expectancy ι was set to 0.2, and probability *p* of transmitting bit “1” varied between 0 and 1.

Parameters of the Ca${}^{2+}$ signaling are listed in [27]: ratio of ER to cytoplasm $a_{1}$ was 0.185, the maximum released flux rate from ER to cytoplasm $v_{chan}$ was 6 s${}^{-1}$, the maximum leaked flux rate from ER to cytoplasm $v_{leak}$ was 0.11 s${}^{-1}$, the maximum pumped flux rate from cytoplasm to ER $v_{pump}$ was 0.9 μMs${}^{-1}$, $b_{1}$ was 0.13 μM, $b_{2}$ was 0.08234 μM, $b_{3}$ was 0.1 μM, $b_{4}$ was 0.9434 μM, $b_{5}$ was 1.049 μM, $b_{6}$ was 0.2 μM${}^{-1}$s${}^{-1}$, diffusion coefficient $D_{Ca}$ was 20 $\mu^{2}$s${}^{-1}$, the average propagation distance of Ca${}^{2+}$ signaling $d_{ER,B}$ was 10 μm, and the length of time slot *T* varied from 1 to 20 s.

Parameters of neural signaling is listed in [15,36,38]: firing rate $\bar{\lambda }$ of neural signaling varied from 15 to 30 Hz, the average release probability of vesicles $p_{v}$ was set to 0.4, pool size of readily release pool $N_{RRP}$ varied from 3 to 10, the pool size of reserve pool $N_{RP}$ was set to 60, and the average filling time $\tau_{f}$ varied from 10 to 40 ms. In axonal transmission, membrane resistance $\gamma_{m}$ was set as 64.1 MΩ, longitudinal resistance $\gamma_{l}$ was set as 8 MΩ, and capacitances $\gamma_{c}$ was set to 1 mF/cm${}^{2}$, the average length of axons $L_{N}$ was set to 43.2 μm, and the length of time slot *T* varied from 1 to 20 s.

### 8.2. Behaviors of Signals

**Figure 4:**
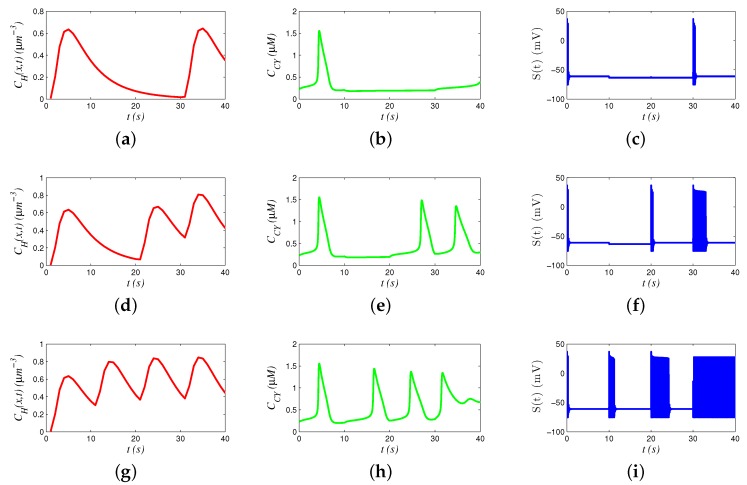
Behavior of signals: (**a**) hormonal signals for “1001”; (**b**) Ca${}^{2+}$ signals for “1001”; (**c**) neural signals for “1001”; (**d**) hormonal signals for “1011”; (**e**) Ca${}^{2+}$ signals for “1011”; (**f**) neural signals for “1011”; (**g**) hormonal signals for “1111”; (**h**) Ca${}^{2+}$ signals for “1111”; and (**i**) neural signals for “1111”.

In the simulation, we present how various signals behave in 40 s to transmit 4 bits of binary information. Figure 4a,d,g presents hormonal signals, Figure 4b,e,h presents Ca${}^{2+}$ signals, and Figure 4c,f,i presents neural signals. The results reveal that the hormonal and Ca${}^{2+}$ signals propagate with low frequency, and the neural signals propagate with high frequency.

We analyzed the chain models by comparing a group of different signals. For example, the signals in Figure 4d,e,f transmit “1011”. Hormone concentrations generate three waves during 0–10 s and 20–40 s. Accordingly, oscillation of Ca${}^{2+}$ concentration is triggered to generate periodic waves. However, oscillation frequency of 20–30 s is smaller than that of 30–40 s, because a part of hormones during 20–30 s still remain in 30–40 s (i.e., memory effect), increasing the hormonal concentration in 30–40 s and promoting the oscillation of Ca${}^{2+}$. Similarly, a faster oscillation of Ca${}^{2+}$ generates more action potential spikes during 30–40 s, compared with those in 20–30 s.

We analyzed the behaviors of various signals by sending different bits. Comparing Figure 4a,d,g, we see that concentrations of hormones are elevated by the percentage of sending bit “1”. Similarly, the frequency of Ca${}^{2+}$ oscillation is also related to percentage of sending bit “1”. In Figure 4h, there are totally four peaks, and the intervals between different peaks are decreasing, since the frequency of Ca${}^{2+}$ oscillation is regulated. Moreover, Ca${}^{2+}$ oscillation stops after 30 s, when hormonal concentration is too high, and the concentration of Ca${}^{2+}$ stabilizes at a high level. In addition, the number of action potential spikes seems positively related to the oscillation frequency of Ca${}^{2+}$.

### 8.3. Channel Capacity

Channel capacity of the proposed communication system is jointly determined by the three types of signaling. Our target was to check the relations between channel parameters and the channel capacity.

**Figure 5:**
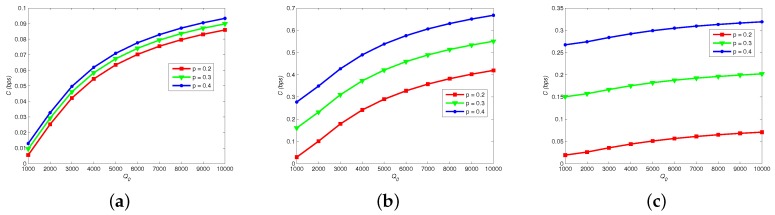
Channel capacity vs. neural firing rate, number of released molecules and probability of transmitting “1”. $N_{RRP}=8$, $\tau_{f}$ = 30 ms, $M_{A}=3$: (**a**) $\bar{\lambda }$ = 20; (**b**) $\bar{\lambda }$ = 25; and (**c**) $\bar{\lambda }$ = 30.

Figure 5 shows how channel capacity *C* changes with different $Q_{0}$, *p* and λ. Apparently, *C* is elevated by increasing number of released molecules $Q_{0}$, because that astrocytes can easily absorb hormones due to the increase of $Q_{0}$ and the error probability of hormonal signaling is decreased. In addition, *C* reaches its peak if *p* is closer to 0.5. By comparing Figure 5a–c, we note that increase of firing rate λ of neural signaling restrains *C*, because a larger λ increases the error probability of vesicle release process.

**Figure 6:**
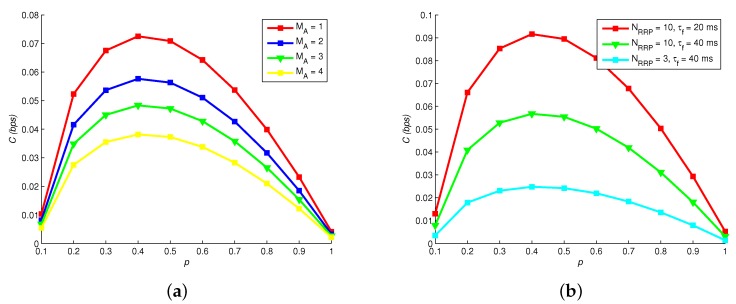
Channel capacity versus: (**a**) astrocytes numbers; and (**b**) vesicle pool models.

Figure 6 shows how channel capacity *C* changes with different $M_{A}$, *p*, $N_{RRP}$ and $\tau_{f}$. Obviously, the optimal *p* of maximizing *C* is not 0.5, which is different with traditional wireless communication. Thereby, the source coding of MC should be particularly designed. Increasing the number of astrocytes $M_{A}$ also elevates *C*, since the relaying capability of Ca${}^{2+}$ signaling is enhanced. Moreover, Figure 6b reveals that *C* is larger with a larger readily release pool $N_{RRP}$ and a shorter filling time $\tau_{f}$.

### 8.4. Transmission Delay

**Figure 7:**
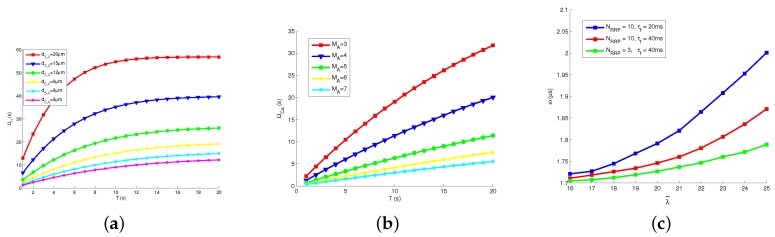
Transmission delay: (**a**) delay of hormonal signaling vs. time slot length and distance; (**b**) delay of Ca${}^{2+}$ signaling vs. number of supporting astrocytes and time slot length; and (**c**) delay of neural signaling vs. pool models.

We checked the relations between various channel parameters and the transmission delay. Figure 7a presents how transmission delay of hormonal signaling $\Omega_{H}$ changes with different *T* and $d_{S,A}$. Apparently, increasing the length of time slot *T* results in the growing $\Omega_{H}$, because releasing rate is decreased by increasing *T* and a fixed number of molecules $Q_{0}$ is released for each bit. The increasing rate of $\Omega_{H}$ degrades fast when *T* is large. Therefore, we deduce that it is important to choose a proper *T* in the proposed model. Besides, we note that $\Omega_{H}$ increases with growing distance $d_{S,A}$ between astrocyte and Tx, since the molecular concentration degrades significantly on the spatial scale.

Figure 7b presents how $\Omega_{Ca}$ changes with different *T* and $M_{A}$. Similar with $\Omega_{H}$, the increased length of time slot *T* signifies the increased $\Omega_{Ca}$. We guess that the variation of *T* impacts the hormonal signaling and Ca${}^{2+}$ signaling. Moreover, a larger $M_{A}$ results in a smaller $\Omega_{Ca}$, because the proposed system could transmit more Ca${}^{2+}$ in a unit time, with the stronger relaying ability.

Figure 7c presents how the average waiting time ω in neural signaling changes with different $N_{RRP}$, $\tau_{f}$ and $\bar{\lambda }$. The values of ω is much smaller than $\Omega_{Ca}$ and $\Omega_{H}$. We see that ω increases with firing rate λ, because firing rate promotes the vesicle release process, more neuro-transmitters are blocked in gap junctions, and ω increases. In addition, ω grows with the increasing size of readily release pool $N_{RRP}$ and the decreasing filling time $\tau_{f}$, since vesicle release process is promoted by those parameters.

## 9. Conclusions

In this paper, we design a chain model for molecular communication to understand and interpret neural signaling. The proposed model contains three types of signals, where Ca${}^{2+}$ signals of astrocytes function as the relay of communication. Due to this advantage, the proposed model becomes more implementable. Behaviors of the different signals demonstrate their close relations with each other. Simulation results validate the effectiveness of the proposed chain model, and also reveal that proper source encoding helps improve the system performance.

Our work provides a philosophy of exploiting complex biological reactions for communication engineering in body area network. Future work will address the expansion of chain modeling and molecular communications in more complex networks, and explore more practical and complex biological activities.
